# Condensed and Dried Milk and Tuberculosis

**Published:** 1915-04-10

**Authors:** 


### CONDENSED AND DRIED MILK AND TUBERCULOSIS.

### The Infectivity of Preserved Specimens.

In calling attention to the recently issued Local Government Board Report, by Dr. Sheridan Delépine, upon the effects of certain condensing and drying processes used in the preservation of milk* we need do no more than remark that the value and interest of the observations and experiments recorded depend upon (1) the frequent presence of bovine tubercle bacilli in milk, and (2) the increasing use of condensed and dried milk.

As we have no efficient milk substitute and as milk so rapidly deteriorates it is not surprising that, amongst the many insidious dangers in our food-supply, the substitution of "preserved" for fresh milk, either habitually or in case of emergency, should have become so common. The methods of condensing or drying milk, although the former process has long been carried on abroad, are not widely understood in this country. Those referred to in the Report are three, which may be very briefly described.

*A. Manufacture of Sweetened Condensed Milk.*—The milk is tested, strained, weighed, mixed in bulk, and rapidly heated, either in steam-jacketed cylinders or in a tubular apparatus, to a temperature of 75° C., 80° C., or even more, being meanwhile stirred or kept flowing. After this "pasteurisation" the milk is further heated to 92° C., and is then mixed with a solution of cane-sugar and transferred to an evaporating vacuum pan where, under a vacuum equal to 65 or 70 centimetres, ebullition occurs at a temperature of 40° to 45° C.; therein the bulk is reduced to about one-third of its original volume. The preparation is then cooled, strained, and tinned.

*B. Drying of Milk over Heated Revolving Cylinders.*—Fresh, weighed milk is allowed to flow regularly on to the surfaces of two steam-heated cylinders, placed side by side and separated by a space of not more than two millimetres, revolving in opposite directions. The milk in the gutter between the cylinders undergoes ebullition, reaching a temperature of about 99° C., and the pellicle of dried milk which forms upon the cylinders is separated by blades in ribbon form, is dried, milled to powder, screened and packed. This method may be applied either to whole or separated milk.

*C. Drying by Spraying Milk into a Current of Hot Air.*—Strained and weighed milk is mixed, warmed, separated if necessary, and pasteurised at a temperature of 70° or 75° C. It is then reduced, in a steam-heated vacuum pan, at a temperature of about 58° C. (raised rapidly in the course of the process to 95° C.), to about one-half of its original bulk. The milk thus condensed is forced by a high-pressure pump, through a spraying-cone in the centre of an opening to which dry air heated to 115° C. is brought, into a tin-lined chamber in which the dried milk falls in granules. The milk powder so produced is screened and packed.

* *Reports to the Local Government Board on Public Health and Medical Subjects.* (New Series, No. 97.) (Food Reports, No. 21.)

The main objects of the inquiry were to ascertain whether tuberculous cows' milk was still capable of conveying tuberculosis after being treated by methods A and B, and the general effects upon the bacterial contents of the milk of methods B and C. It is impossible here to refer at length to the experimental details, but one important observation, although a side issue, cannot be passed by. The question of the infectivity of the milk of cows suffering from tuberculosis other than tuberculous disease of the udder has been frequently discussed. Dr. Delépine cites a case of tuberculous disease of the left anterior quarter of the udder of a cow, the disease spreading gradually to the right anterior and left posterior quarters; whilst the milk from these quarters contained tubercle bacilli, the milk from the right posterior quarter was proved, by frequent examinations, to have remained, during a whole month, free. The fact that a healthy quarter of the udder of a tuberculous cow may yield milk free from infection supports the view that tubercle bacilli are not frequently (if ever) secreted in a sound udder.

The conclusions arrived at show that certain bacteria may remain alive for a considerable time in sweetened condensed milk, although they do not usually multiply in it in the condensed and tinned condition. The total number of bacteria present in milk, such as is usually supplied to town consumers, is considerably reduced by treatment according to each of the three methods investigated; the reduction was greatest in the case of method A. "The distribution of bacteria in the milk before, during, and after treatment indicated clearly that many of them which had disappeared while the milk was heated and protected from the access of dust and from contact with contaminated articles, had reappeared in the final stages of preparation when no longer protected against contamination. Many of the bacteria found in preserved milk, as found on the market, owe their presence to a process of recontamination." "Both the fresh and the prepared milk derive most of these bacteria from the same sources, which are water, soil, and dirt, acting directly or through the intermediation of air-currents, vessels, persons, etc." At none of the stages of preparation was the milk ever found completely sterile; among the bacteria which were invariably found to resist the so-called "pasteurisation" were those included under the term *Bacillus mesentericus*. Some tubercle bacilli also survived it; Dr. Delépine, by the way, finds reason for disagreeing with many of the published statements concerning the thermal death-point of the tubercle bacillus. "The tubercle bacilli which had survived pasteurisation in method A and drying by heat in method B were still capable of producing progressive tuberculosis in guinea-pigs inoculated subcutaneously with milk containing these bacilli, but the course of the disease produced by these bacilli was very much slower than that of the disease produced in guinea-pigs inoculated with the same amount of untreated tuberculous milk. The tuberculosis produced by the heated bacilli was latent or occult for some four weeks. Young rabbits fed with milk containing these modified bacilli did not contract tuberculosis." None of the sweetened condensed milk prepared from the infective (tuberculous) milk produced tuberculosis in the inoculated animals, but some of the samples caused inflammatory lesions not produced by the pasteurised milk before sweetening and condensing.

